# Supplementary figures and images for: Likelihood-ratio test statistic for the finite-sample case in nonlinear ordinary differential equation models
Source: PLoS Comput Biol. 2023 Sep 22;19(9):e1011417. doi: 10.1371/journal.pcbi.1011417 (PMC10550180; doi:10.1371/journal.pcbi.1011417)

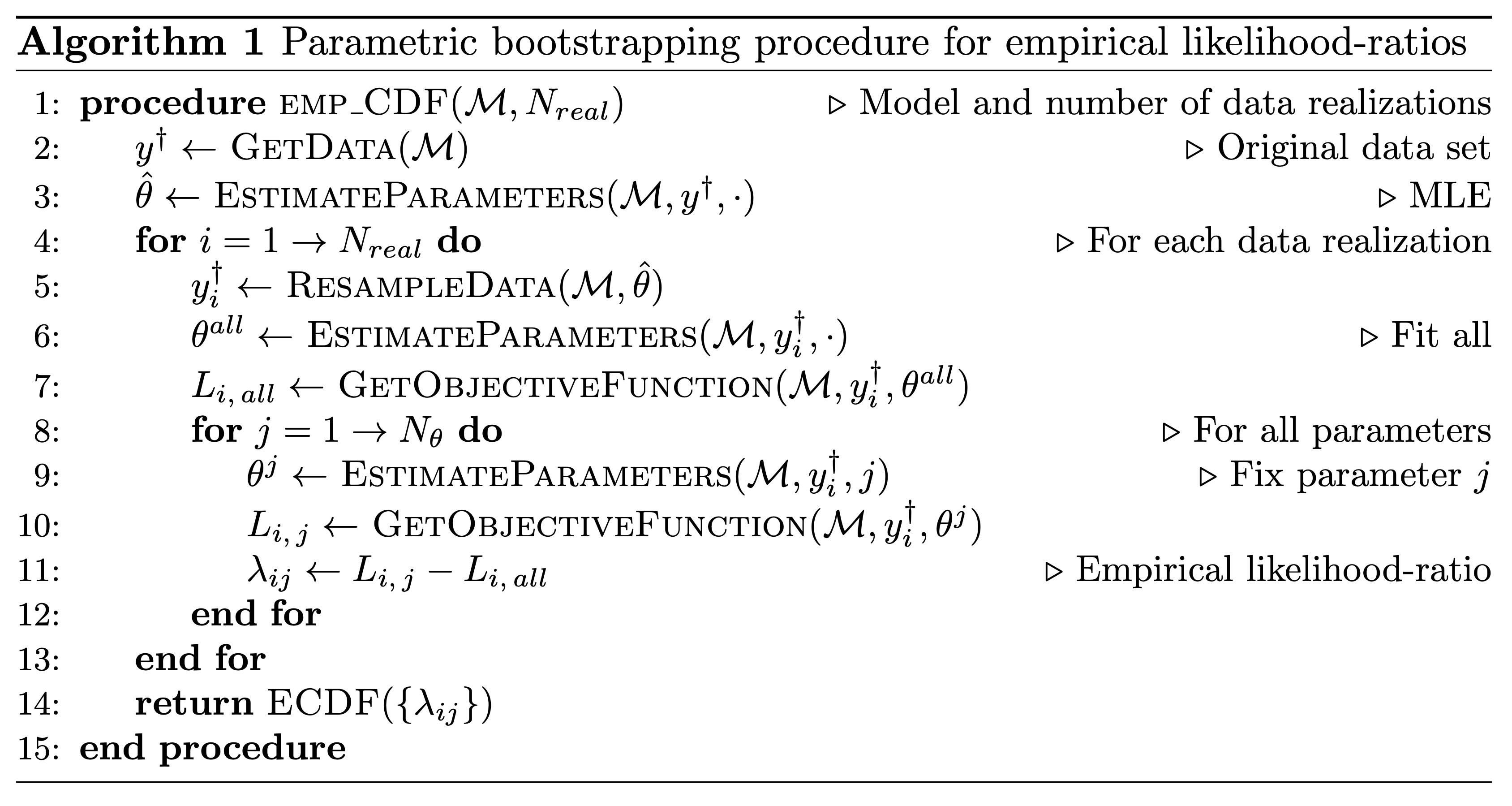

Supplement: S1 Fig — (TIF) [file pcbi.1011417.s001.tif]
